# Does hyperbaric oxygen therapy pressure reduce mechanical stability of implants?

**DOI:** 10.1007/s10856-022-06680-5

**Published:** 2022-07-15

**Authors:** Anıl Özyurt

**Affiliations:** Department of Oral and Maxillofacial Surgery, Faculty of Dentistry, Dokuz Eylül University, İzmir, 35340 Turkey

## Abstract

Hyperbaric oxygen therapy (HBOT) has beneficial effects for patients complaining of poor bone healing such as related to diabetes mellitus. However, it is known that changing pressure conditions might cause dental barotrauma in the oral cavity. The aim of this study was to evaluate implant mechanical stability under HBOT pressure. Thirty-five implants were placed in bone blocks divided into five groups as control, 1, 3, 5, 7 HBOT cycles. In one cycle, 2.4 bar 100% oxygen pressure was performed. Implants’ stabilities were measured with resonance frequency analysis (RFA) and removal torque (RT) meter device. Data were analyzed using Shapiro Wilk, ANOVA, and Tukey HSD tests for RFA and RT values considering *p* < 0.05 as the statistical significance level. RFA and RT values were compared by Pearson correlation coefficiency. RFA values of 5 and 7 HBOT cycles were significantly lower than 1, 3 HBOT and control group (*p* < 0.001). There was no statistical difference between 5 and 7 HBOT cycles RFA values. HBOT pressure simulation slightly but statistically decreased the stability for the implants exposed to 5 and 7 HBOT cycles.

**Figure:**
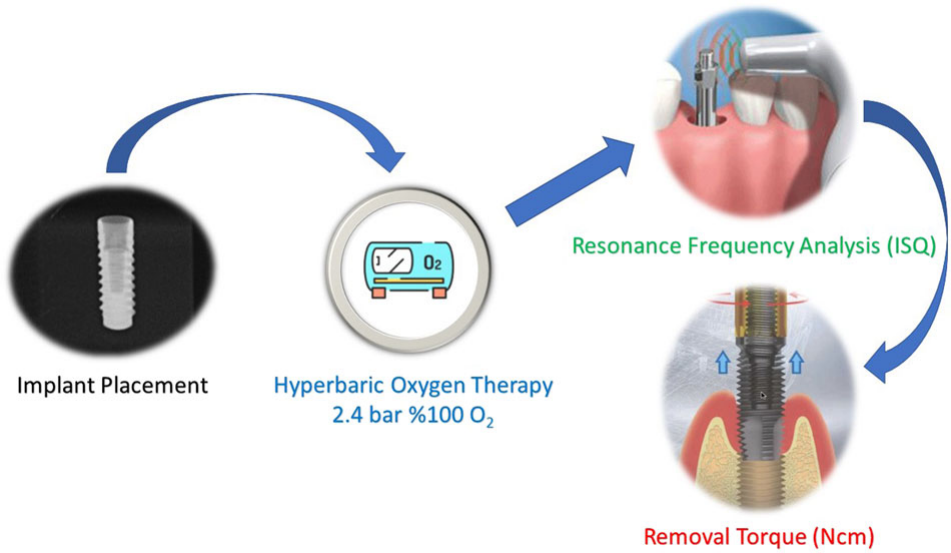
Graphical abstract

Graphical abstract

## Highlights

- The primary stability of an implant is the crucial factor for achieving osseointegration.
- There is no experimental study showing the effect of hyperbaric oxygen therapy (HBOT) pressure on mechanical stability of implants.
- This mechanical study demonstrated 5 and 7 HBOT sessions had a slightly reducing effect on immediate implant stability.

## Introduction

Hyperbaric oxygen therapy (HBOT) is an established treatment option for poor wound healing associated with some diseases such as diabetes mellitus [1]. The patients stay in an ~2.4 bar pressured chamber for a while and inhale 100% oxygen. Thus the oxygen saturation can be increased in the tissue, and the wound healing process may be accelerated [2]. The treatment regimens vary from 30 to 120 min once or twice a day. The number of sessions is determined by a medical director who is specialized in hyperbaric medicine. The medical director monitors patients’ vitals during the session because the pressured 100% oxygen might poison the individuals. Also, the chamber’s changing pressure might cause dental barotrauma. Therefore, the compressing and decompressing procedures must be performed slowly [3].

While primary stability is decreasing, secondary stability is increasing thanks to new bone regeneration. Three weeks after implant placement, the stability would be the intersection point of primary and secondary stability as the lowest value [4]. So first three weeks are more critical in terms of soft tissue healing and protecting the implant from external forces.

Resonance frequency analysis (RFA) is a reliable methodfor evaluating the stability of implants. RFA is specialized only for implants [5]. Besides, removal torque (RT) value is used as a stability marker in in-vitro implant studies [6].

Although it is known that air pressure can affect dental tissues, there is limited evidence-based information on oral complications in the literature. Zadik and Drucker explained that diving hyperbaric conditions could cause dental complications [7]. Peker et al. reported a treatment case about tooth fracture caused by diving under 35 m [8].

Implant patients may be exposed to hyperbaric environments like underwater, quarry, mine, or HBOT. Implant patients might avoid high-level hyperbaric work areas during the implant healing process, but HBOT can be necessary and might not be stopped for treating severe diseases, or HBOT might also have beneficial effects on bone healing. However, Özyurt reported that changing pressure caused a loosening effect on the internal screw of implants [9]. Therefore the possible negative mechanical effects of changing hyperbaric conditions must be tested on implants with in vitro conditions first. The aim of this study was to evaluate if there is a negative mechanical effect on implant stability or not under HBOT pressure. The null hypothesis tested was that hyperbaric conditions would not affect implant mechanical stability.

## Materials and methods

### Implant system and sample preparation

**Fig. 1:**
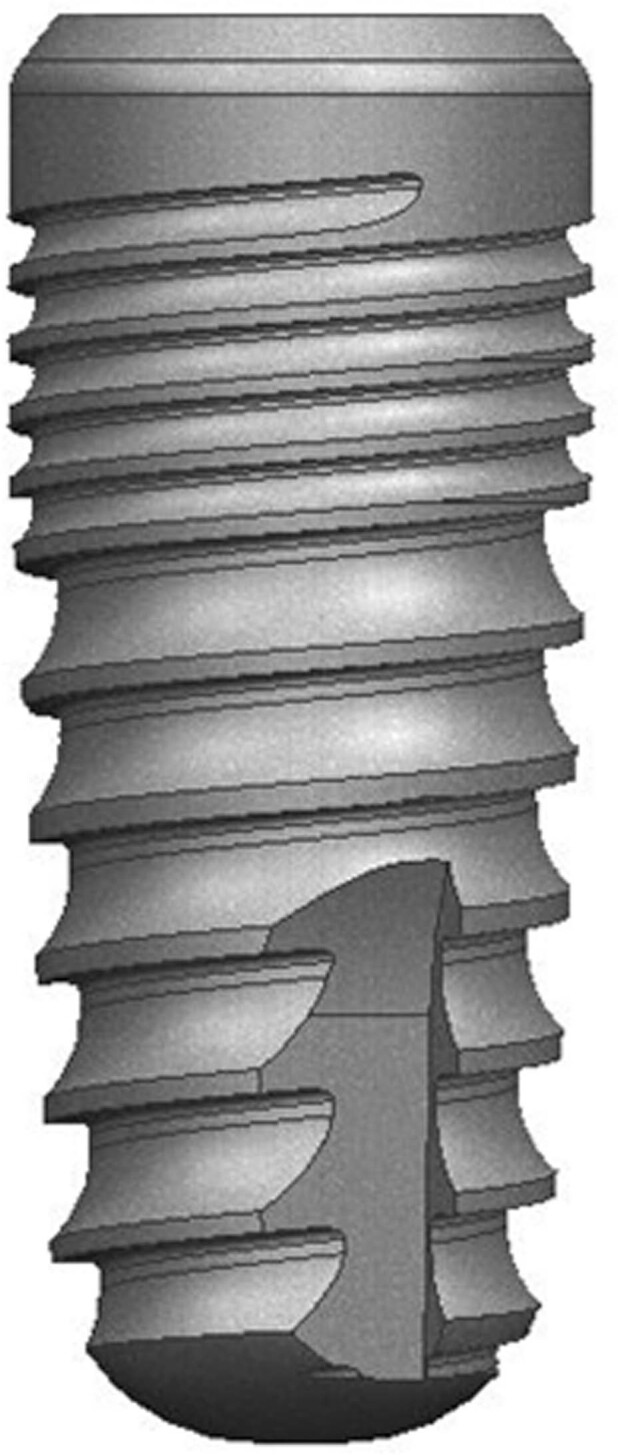
Schematic drawing of the dental implant: 11-degree tapered and bone level (BioInfinity, Avrupa Implant Sanayi ve Dış Ticaret Ltd., Istanbul, Turkey)

**Fig. 2:**
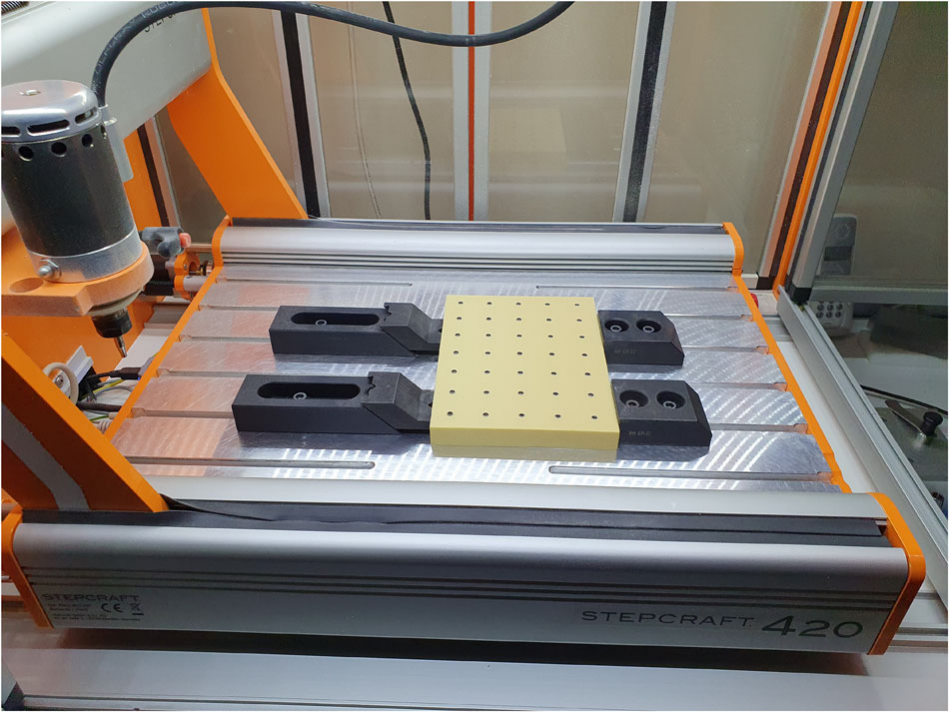
Implants were placed in Sawbones bone simulation block by Stepcraft 420 computer-aided drilling device (Stepcraft GmbH & Co. KG, Menden, Germany)

An independent statistician reviewed the methodology for the sample size before the study. Power analysis was used to determine the reliability of results by taking the probability of 0.05 error (Type I) and intra-class correlation coefficient 0.90 with 80% power. Thirty-five tapered bone-level implants (BioInfinity, Avrupa İmplant Sanayi ve Dış Ticaret Ltd., Istanbul, Turkey), measuring 4.2 mm in diameter and 10 mm in length, were used (Fig. 1). All implant components were made of Ti-Gr 23 (Titanium Grade 5 ELI; Ti6Al4V). Implant cavities were prepared in Sawbones homogenous bone simulation blocks (A Pacific Research Company, Vashon Island, Washington, USA) by Stepcraft 420 computer-aided drilling device (Stepcraft GmbH & Co. KG, Menden, Germany) with standard protocol drilling protocol using new drills, and the implants were placed with a 35 N cm static torque force according to the manufacturer’s instructions (Fig. 2). Sawbones block had 20 PCF densities made of solid closed-cell wall foam that simulated the cancellous jawbone. Implants were placed at 1.5 cm distances from each other, and the main block was divided into five equal rectangular prisms, which imitated jaw bone shape. Cover screws were fixed into all implants before hyperbaric application.

### Hyperbaric oxygen therapy pressure application

**Fig. 3:**
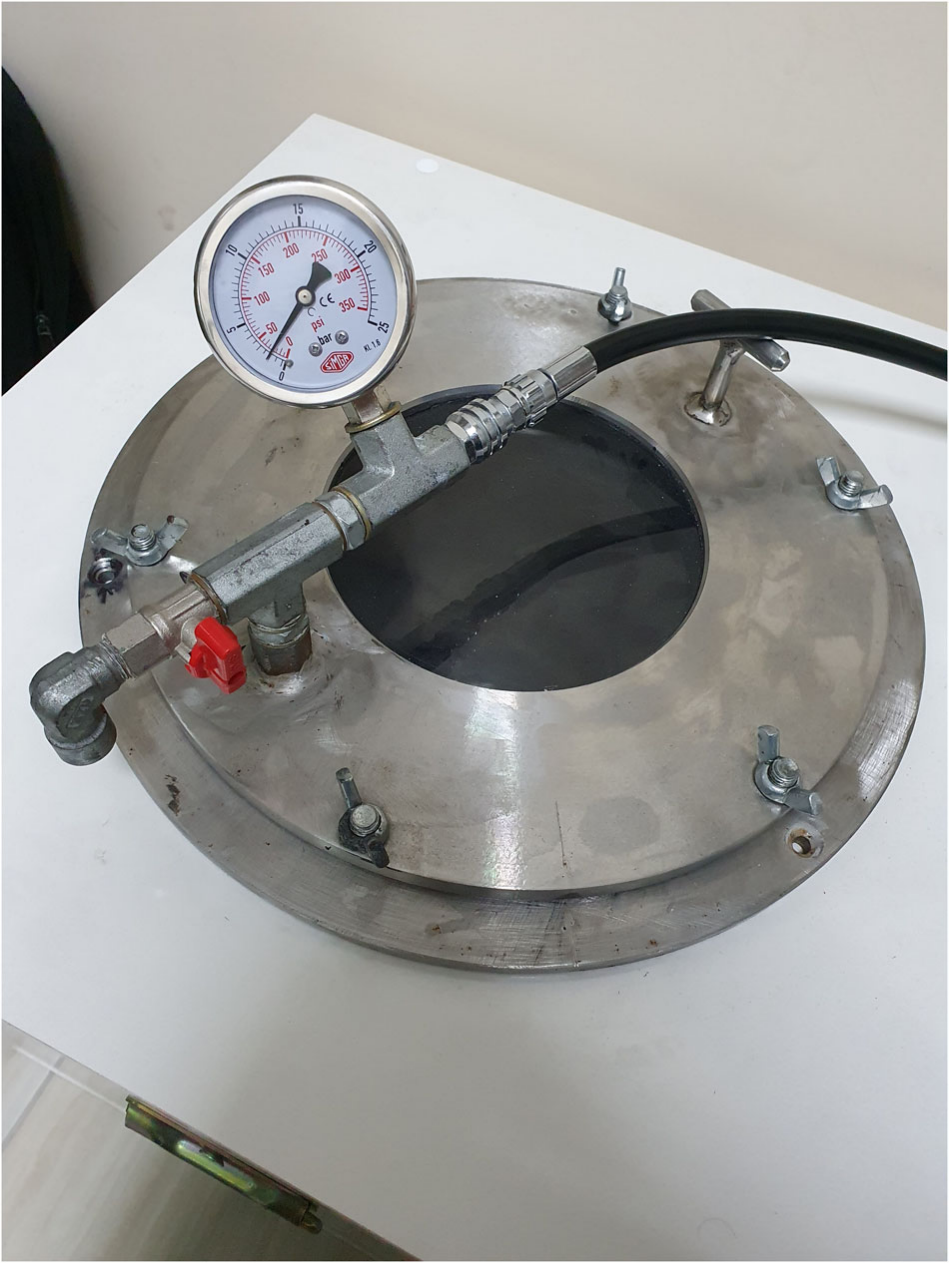
10-l steel pressure vessel for HBOT pressure application

All implants were divided into five groups (*n* = 7) as one control (C) and four experimental groups (H_I_, H_III_, H_V_, H_VII_). The experimental groups were exposed to the hyperbaric pressure in a 10-l steel pressure vessel, respectively 1, 3, 5, and 7 cycles which simulated seven HBOT session days. In one HBOT session, 2.4 bar pressure (by obeying compression–decompression period protocol: 15 min for compression and 15 min for decompression) was applied for a total of 1 h per day [10]. Two precalibrated gauges were used to monitor the pressure in the vessel. Gas flow (100% oxygen) was transferred constantly from a high-pressure aluminum tank connected to a pressure regulator device (Fig. 3). No pressure procedure was applied to the control group.

### Resonance frequency analysis and removal torque measurements

**Fig. 4:**
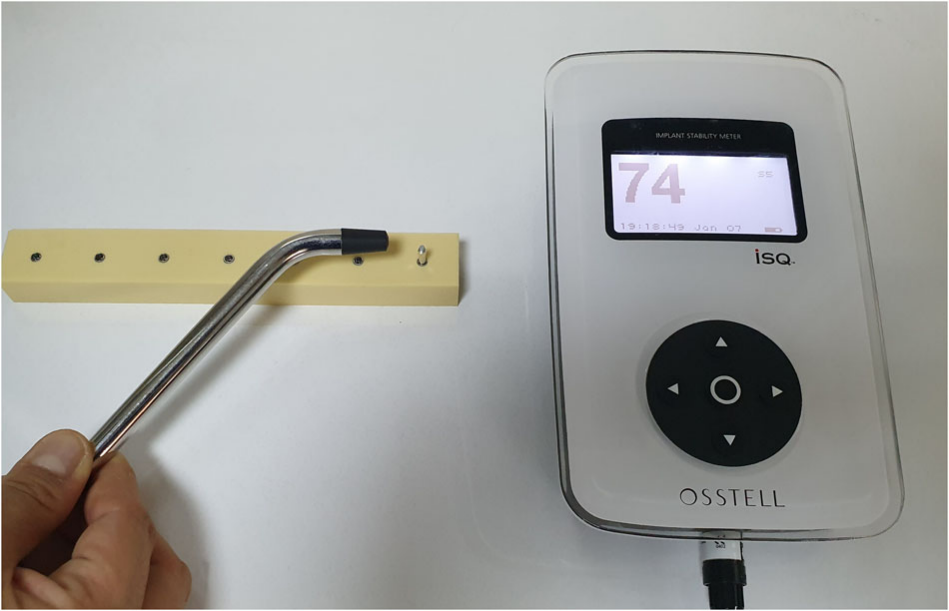
Osstell RFA device (Osstell, Gothenburg, Sweden) for measuring ISQ

Resonance frequency analysis (RFA) was performed before and after the HBOT pressure application using the Osstell ISQ device (Osstell, Gothenburg, Sweden). Initial RFA measurement was performed before HBOT sessions, second RFA measurement was performed after HBOT sessions on the seventh day. Type 54 SmartPegs (Osstell, Gothenburg, Sweden) were connected to implants with finger-tightening force. All implants were measured from four directions, and the mode ISQ (Implant Stability Quotient) value was recorded for each implant (Fig. 4).

**Fig. 5:**
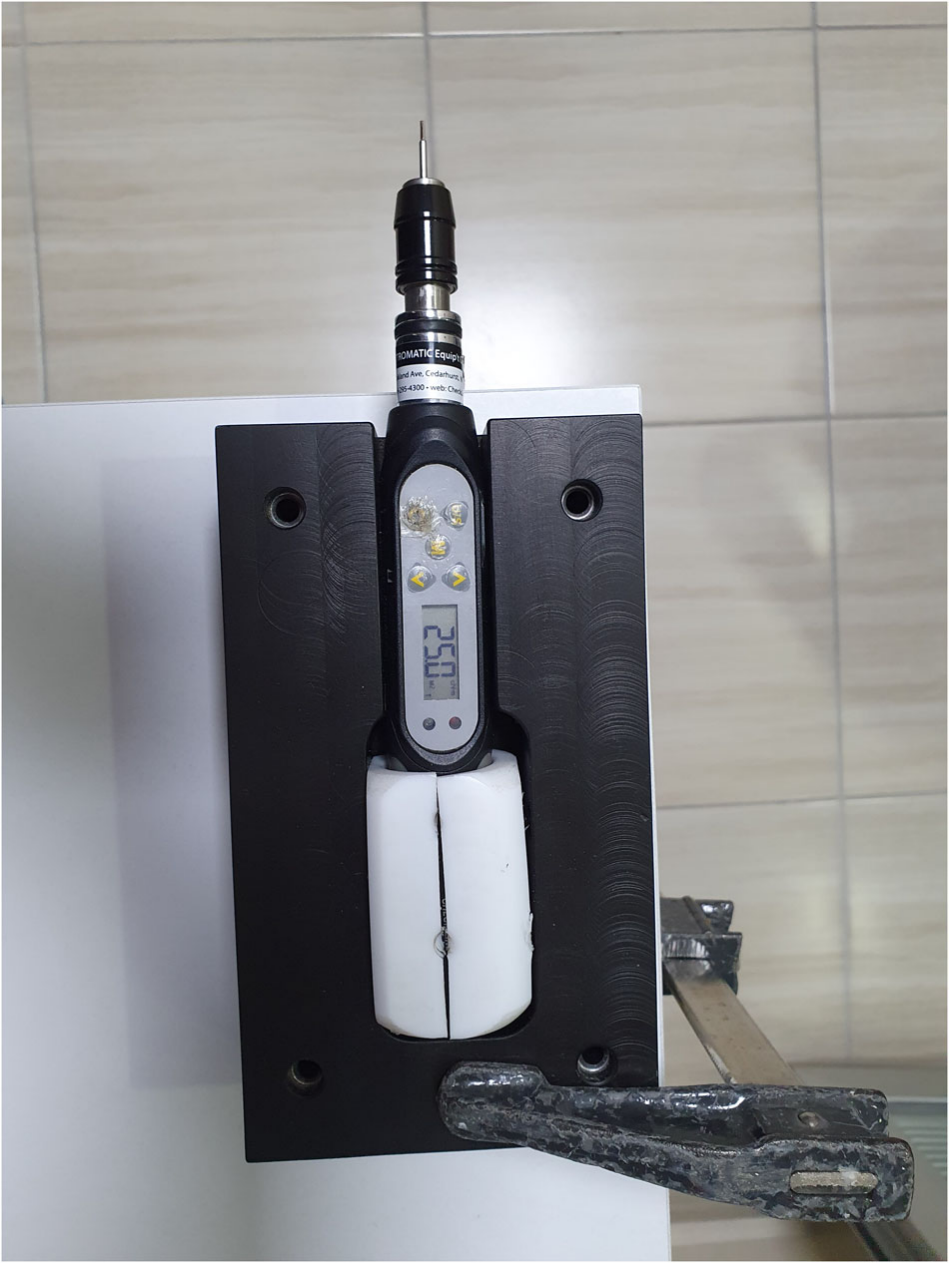
TSD digital torque meter device (Electromatic Equipment Co. Inc., Cedarhurst, USA) for measuring removal torque

After the second RFA performing, the RT were measured using a TSD digital torque meter (Electromatic Equipment Co. Inc., Cedarhurst, NY, USA) and a manual rotation arm (Fig. 5). The arm applied rotation force slowly counter-clockwise. The minimum torque force able to untighten the implant was automatically recorded as the Ncm unit by the torque meter. All samples were measured, respectively.

### Statistical analysis

RFA and RT data were analyzed using IBM SPSS Statistics for Windows, version 23.0 (IBM Corp., Armonk, NY, USA) by an independent statistician. Shapiro-Wilk tests were performed to determine if the data revealed a normal distribution. The data were distributed normally. ANOVA and Tukey HSD tests were used to evaluate for inital RFA, second RFA, and RT. Besides, the Pearson correlation coefficient was used for determining the relation between second RFA and RT values. Mean ± standard deviation and median (min–max) values were presented (accepted statistical significance level was *p* < 0.05).

## Results

### RFA findings

There were no statistical differences among the control, 1, 3, 5, and 7 HBOT groups (*p* > 0.05) for the initial RFA measurements (Table 1).

**Table 1:** Comparison of groups for initial RFA values

	Implant stability (ISQ), Initial RFA	
	Mean value ± Standard deviation	Median(min.–max)
*C*_initial_	74,14 ± 0,69	74 (73–75)
H_I_	74,29 ± 0,76	74 (73–75)
H_III_	74,14 ± 0,69	74 (73–75)
H_V_	73,71 ± 0,76	74 (73–75)
H_VII_	73,71 ± 0,76	74 (73–75)
ANOVA statistics		*F* = 0938
*p*		**0.456**

*F* ANOVA analysis test statistic

There were statistical differences among the control, 1, 3, 5, and 7 HBOT groups (*p* < 0.001) for the second RFA measurements. Group H_VII_ and Group H_V_ showed lower mean ISQ values than Group C, Group H_I,_ and Group H_III_. However, there were no statistical differences between Group H_V_ and Group H_VII_; therewithal among Group C, Group H_I,_ and Group H_III_ (Table 2).

**Table 2:** Comparison of groups for second RFA and RT values

	Implant Stability (ISQ), Second RFA		Removal torque (Ncm)	
	Mean value ± Standard deviation	Median (min.–max)	Mean value ± Standard deviation	Median (min.–max)
C_end_	72,9 ± 0,7^a^	73 (72–74)	33,7 ± 0,3^c^	33,8 (33,2–34,1)
H_I_	72,7 ± 0,8^a^	73 (72–74)	33,6 ± 0,3^c^	33,7 (33,2–34)
H_III_	72,9 ± 0,7^a^	73 (72–74)	33,7 ± 0,3^c^	33,6 (33,4–34,2)
H_V_	69,7 ± 0,5^b^	70 (69–70)	32,7 ± 0,2^a^	32,7 (32,4–32,8)
H_VII_	68,9 ± 0,7^b^	69 (68–70)	32,2 ± 0,2^b^	32,1 (32–32,7)
Test statistics	F = 59,745		*F* = 49,065	
*p*	**<0.001**		**<0.001**	

*F* ANOVA analysis test statistic

^a–c^No statistically significant difference exists between columns marked with the same superscript letters

When Table 1 and Table 2 were compared, there was a decrease in all implants’ RFA values.

### RT findings

There were statistical differences among the control, 1, 3, 5, and 7 HBOT groups (*p* < 0.001). Group H_VII_ and Group H_V_ showed lower mean Ncm values than Group C, Group H_I,_ and Group H_III_. However, Group H_V_ showed a significantly higher RT value than Group H_VII._ There were no statistical differences among Group C, Group H_I,_ and Group H_III_ (Table 2).

### Second RFA and RT correlation findings

**Fig. 6:**
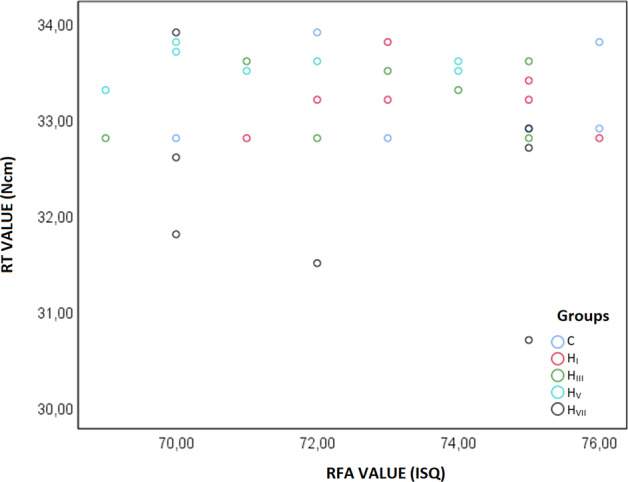
Scatter plot of ISQ and Ncm values

There was a high-level positive statistical correlation between ISQ and Ncm values among all related groups (*r* = 0976, *p* < 0001). Table 3 Pearson coefficient correlation and Fig. 6 showed the scatter plot of ISQ and Ncm values.

**Table 3:** Correlation between second RFA and RT values

Grup			Removal torque (NCM)
Control	Implant Stability (ISQ)	*r*	0.976
		*p*	**<0.001**
H_I_	Implant Stability (ISQ)	*r*	0.896
		*p*	**0.006**
H_III_	Implant Stability (ISQ)	*r*	0.913
		*p*	**0.004**
H_V_	Implant Stability (ISQ)	*r*	0.874
		*p*	**0.010**
H_VII_	Implant Stability (ISQ)	*r*	0.834
		*p*	**0.020**
Total	Implant Stability (ISQ)	*r*	0.985
		*p*	**<0.001**

*r* Pearson correlation coefficiency

## Discussion

The present study is the first experimental research study that evaluated the mechanical implant stability under HBOT pressure conditions. But there are some case reports and various studies about implant under hyperbaric conditions in the literature. Özyurt reported that various air pressure had a loosening effect on internal screws of the implants [9]. Salgado-Peralvo et al. reported a patient whose implant was supposed to fail because of scuba diving [11]. In that case report, the patient performed scuba dives regularly until two months after implant placement, and the implant was exposed to micro-motion related to changing pressure. Brandt stated that pressure might cause dislodgement to the prosthesis, not osseointegrated implants [12]. An in-vitro study conducted by von See et al. showed that changes in atmospheric conditions might decrease osteoblast activity in bone augmentation [13]. In our present study, the HBOT pressure simulation was applied for the first seven days after implant placement; thus, application time was imitated to stay in the mechanical stability period.

Gunepin et al. reported a dental fracture case that was related to biting a scuba mouthpiece [14]. In HBOT, a face mask is used, and there is no intraoral biting piece. Most dental barotrauma cases are related to tooth and prosthesis complications. Zadik and Drucker associated hyperbaric diving conditions with several dental complications [15]. Zadik also concluded that pressure changes in microscopic air bubbles in the cement layer beneath crowns might significantly reduce prosthetic device retention [7]. Peker et al. reported a case of dental restoration dislodgement during scuba-diving at a depth of 35 m [8]. In our study, HBOT pressure was equivalent to a depth of 14 m underwater pressure. An HBOT session may vary from 30–120 min. The compression and decompression procedure is more critical than total procedure time about mechanical effects [16]. In our study, we applied the least HBOT procedure application time and focused more on the changing pressure effect by obeying the decompression and compression speed limits. Our experiment model simulated the proper implant placements for determining the HBOT’s mechanical effect unbiasedly. For optimizing standardization, bone simulation blocks were used [17, 18]. Also, there are cadaver and animal studies for primary stability evaluations in the literature [19, 20]. Implant site preparation directly affects the mechanical implant stability [21]. In our study, recommended regular protocol with a computer-aided driller was used before implant placement. According to the manufacturer’s recommendation, insertion torque was 35 Ncm [22]. Implant shape and threads’ distribution directly affect mechanical stability. Sharp-threaded tapered implants were advised for achieving good mechanical stability for cancellous bone [22–25]. In our study, we preferred to use tapered-form implants for being able to resist hyperbaric pressure forces.

According to our study, 5 and 7 cycles of HBOT pressure application reduced the implant mechanical stability. Therefore the null hypothesis was partially rejected. It was reported that there was a loosening effect on internal screws under hyperbaric conditions [9]. Similarly, repeating compression and decompression forces may affect implant stability.

RFA analysis is the most accepted method for evaluating implant stability [26–29]. RT value is another marker for evaluating stability [30]. Tabassum et al. reported a correlation between RFA and RT values [31, 32]. There was a significant correlation between RFA and RT values in our study and this correlation validated our procedure’s reliability.

This study is an initial experiment and demonstrated the effect of the first HBOT week on implant stability with only mechanical aspects by neglecting cellular activity. Because it is known that if there is mobility, bone healing may be impaired in the ongoing process. Nevertheless, ignoring biological factors is certainly a major limitation of this study. Further experiments and clinical studies on the healing process are needed to determine more reliable results.

## Conclusion

According to the present study;

1. Five and seven HBOT cycles had a slightly but significantly reducing effect on mechanical implant stability.
2. All implants’ RFA values also decreased after 7 days spontaneously.
3. Both RFA and RT evaluations can be reliably used for determining the implant stability. There was a statistically significant correlation between RFA and RT values for evaluating the implant stability thus stability tests were confirmed each other.

## Abbreviations

HBOT: hyperbaric oxygen therapy
RFA: resonance frequency analysis
RT: removal torque
ISQ: implant stability quotient
